# Case Report: Cerebellar microhemorrhages: an underrecognized feature of MMA-HC revealed by high-field 7.0 T MRI

**DOI:** 10.3389/fradi.2025.1654311

**Published:** 2025-10-16

**Authors:** Ye Ran, Wanjun Li, Yunyun Huo, Shengyuan Yu, Zhao Dong, Chenglin Tian

**Affiliations:** 1st Medical Center of Chinese PLA General Hospital, Beijing, China

**Keywords:** methylmalonic acidemia, cerebral microhemorrhages, 7 Tesla MRI, homocystinuria, SWI

## Abstract

Cerebellar microhemorrhages have not been previously documented in methylmalonic acidemia with homocystinuria (MMA-HC), a rare inherited metabolic disorder. Herein, we reported an 18-year-old female presented with acute gait instability and dysarthria post-febrile illness. Biochemical testing revealed severe hyperhomocysteinemia. Brain MRI demonstrated bilateral cerebellar DWI/FLAIR hyperintensities. Whole-exome sequencing confirmed compound heterozygous MMACHC mutations, establishing cblC-type MMA-HC diagnosis. Symptoms resolved after one month of vitamin-based therapy. Follow-up 3.0 T MRI and 7.0 T MRI susceptibility-weighted imaging (SWI) uncovered multiple punctate cerebellar vermian microhemorrhages—a previously unreported finding. This case highlights an unusual adult-onset presentation of MMA-HC and represents the first report of SWI-detectable cerebellar vermis microhemorrhages with this condition, visualized. This finding suggests that cerebellar microhemorrhages may be an under-recognized feature in MMA-HC, particularly detectable using high-field SWI during acute exacerbations, and contributes to a more comprehensive understanding of the neurological complications in this metabolic disorder.

## Introduction

Methylmalonic acidemia (MMA) represents a group of rare, autosomal recessively inherited disorders of organic acid metabolism, primarily caused by defects in methylmalonyl-CoA mutase or its cofactor, cobalamin (vitamin B12) metabolism. This metabolic block leads to the pathological accumulation of methylmalonic acid, propionylcarnitine, and other toxic metabolites, resulting in multisystem dysfunction (1). Classically, MMA manifests in infancy or early childhood with acute life-threatening metabolic crises characterized by encephalopathy, ketoacidosis, hyperammonemia, and developmental delay (2). While predominantly a pediatric disorder, rare late-onset forms presenting in adolescence or adulthood are increasingly recognized, often with distinct neurological or psychiatric features.

A significant subtype is cobalamin C (cblC) deficiency, caused by mutations in the MMACHC gene. This specific defect impairs the conversion of dietary cobalamin into its active forms, adenosylcobalamin (AdoCbl) and methylcobalamin (MeCbl), leading to combined methylmalonic acidemia and hyperhomocysteinemia (MMA-HC). MMA-HC is the most common form of MMA in China (3). Neurological manifestations are a hallmark of late-onset MMA-HC and can include cognitive decline, psychiatric disturbances, myelopathy, peripheral neuropathy, and thromboembolic events. Neuroimaging findings are variable but commonly include cerebral atrophy, white matter abnormalities, and basal ganglia lesions (4, 5). Cerebellar involvement, while recognized as a significant feature in some cases, is less frequently reported as the dominant presentation.

Notably, neuroimaging descriptions of cerebellar pathology in MMA-HC have primarily focused on T2/FLAIR hyperintensities or atrophy (6, 7). Secondary hemorrhagic complications within the central nervous system in MMA are exceedingly rare and have been reported almost exclusively in infants during acute metabolic decompensation, often associated with catastrophic outcomes (8). Crucially, cerebellar microhemorrhages have never been documented in adolescent or adult-onset MMA-HC patients, nor have they been described as a sequela detected during recovery.

This case report describes an 18-year-old female with genetically confirmed late-onset MMA-HC who presented with acute cerebellar syndrome following a febrile illness. While bilateral cerebellar signal abnormalities were evident acutely, the novel and pivotal finding was the subsequent detection of multiple punctate microhemorrhages confined to the cerebellar vermis using high-field susceptibility-weighted imaging (SWI) during clinical recovery. This represents the first report of SWI-detectable cerebellar microhemorrhages in an adolescent with MMA-HC, highlighting a previously unrecognized potential neuropathological consequence of this metabolic disorder and underscoring the utility of advanced MRI techniques in elucidating its full spectrum**.**

## Case report

An 18-year-old female presented with 26 days of progressive gait instability, dysarthria, and limb weakness. Symptoms were preceded by a 4-day high fever (39°C) with dizziness, headache, and vomiting. Past medical history was unremarkable. She had average academic performance since childhood and normal motor development. Family history was non-contributory. Neurological examination revealed: mildly impaired finger-to-nose test bilaterally, mildly impaired heel-to-shin test bilaterally, inability to walk in a straight line.

**Figure 1:**
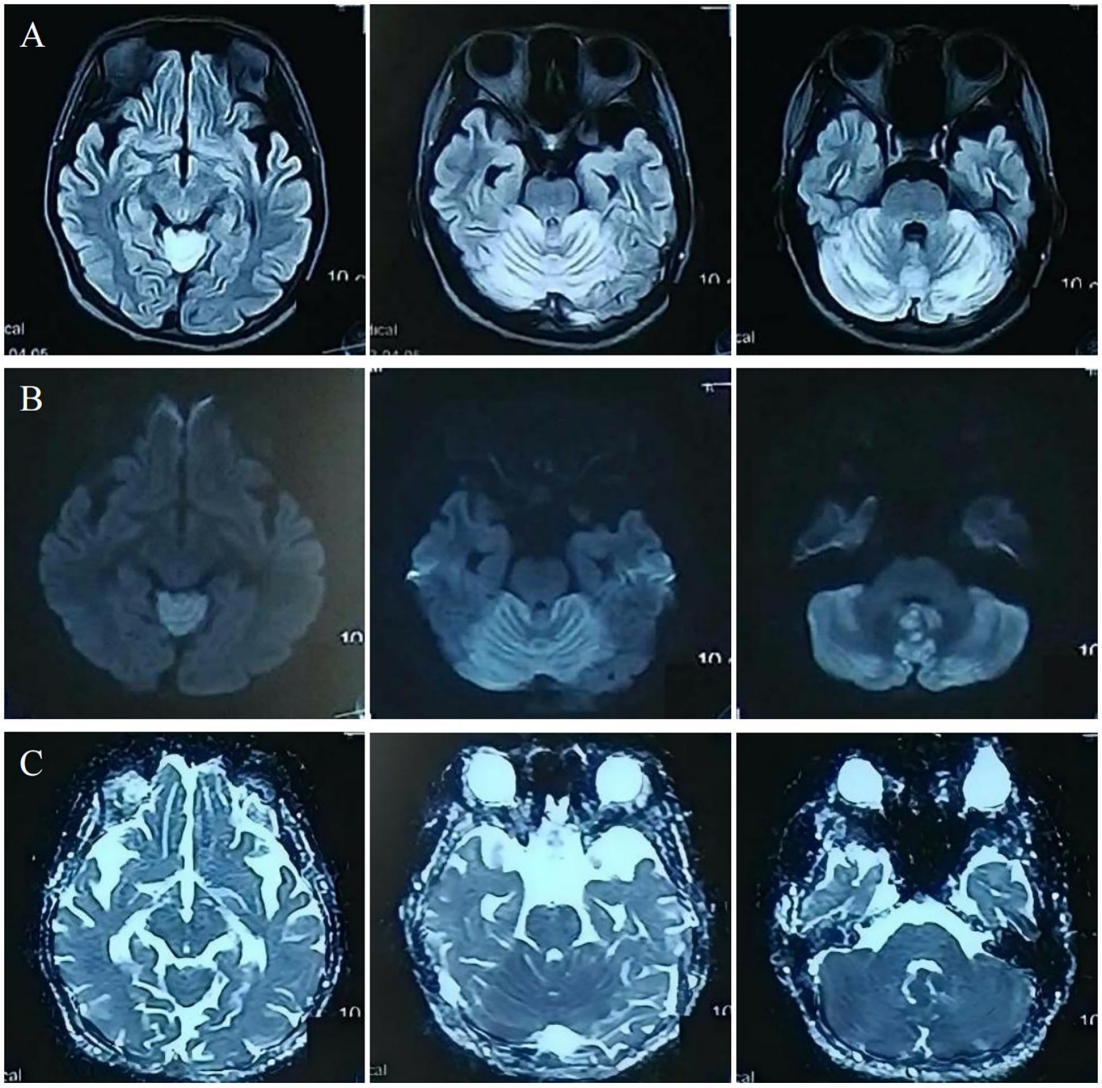
Acute MRI: cerebellar hyperintensities on FLAIR **(A)**/DWI **(B)**; no ADC abnormality **(C).**

Investigations revealed severe anemia (hemoglobin 93 g/L, ref: 114–154 g/L) and hyperhomocysteinemia (210 µmol/L, ref: ≤15 µmol/L). Laboratory investigations also revealed an elevated blood propionylcarnitine/acetylcarnitine ratio (C3/C2: 0.21; ref: 0.02–0.20) and significantly increased urinary methylmalonic acid (17.5; ref: 0.0–4.0) with mild methylcitric acid elevation (0.8; ref: 0.0–0.7) on tandem mass spectrometry and organic acid analyses. Cranial MRI showed bilateral cerebellar hyperintensities on MRI DWI/FLAIR (Figure 1). Whole-exome sequencing identified compound heterozygous MMACHC mutations (c.80A > G, c.482G > A), confirming a diagnosis of cblC-type MMA-HC.

**Figure 2:**
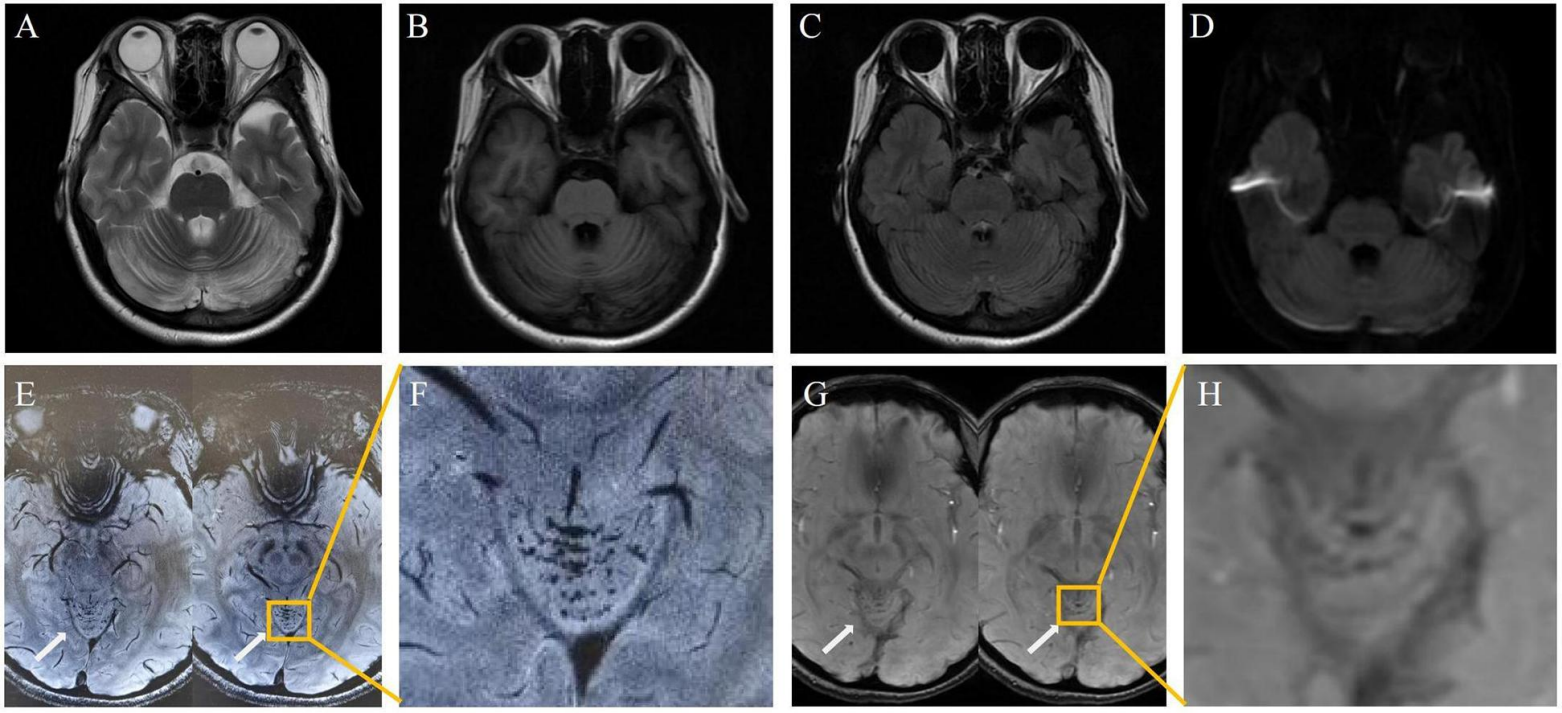
Post-treatment: cerebellar lesion resolution [**(A–D)**, 3.0 T]; vermian microhemorrhages on 7.0 T SWI **(E,F)** and 3.0 T SWI **(G,H)**.

The symptoms resolved gradually following a one-month course of treatment, which included intramuscular injection of mecobalamin (500 μg once daily), as well as oral administration of vitamin B6 (10 mg three times daily), folic acid (5 mg once daily), and idebenone (30 mg three times daily). Follow-up 3.0 T MRI showed significant resolution of the cerebellar lesions on DWI and FLAIR (Figure 2A–D). Crucially, 7.0 T and 3.0 T MRI SWI revealed multiple punctate microhemorrhages in the cerebellar vermis (Figure 2E–H). The long-term treatment plan for the patient consists of lifelong medication including intramuscular injection of mecobalamin 500 μg once daily—to be reduced to once weekly after homocysteine levels stabilize—along with oral folic acid 5 mg once daily, and oral vitamin B6 10 mg three times per day. In addition, regular monitoring of plasma homocysteine, folate levels, and renal function is recommended, with dosage adjustments made based on the results.

## Discussion

This case presents three notable features in an adolescent with late-onset MMA-HC: (1) acute cerebellar syndrome as the dominant presentation; (2) bilateral cerebellar diffusion-restricting lesions during acute exacerbation; and (3) the novel detection of cerebellar vermis microhemorrhages during recovery using high-field SWI MRI. While cerebellar involvement (e.g., atrophy, signal abnormalities) is recognized in MMA, microhemorrhages—particularly isolated to the vermis—represent an unreported neuropathological finding in this age group.

The pathogenesis of microhemorrhages in MMA-HC is multifactorial. It involves homocysteine-induced endothelial dysfunction, which promotes oxidative stress—including endothelial nitric oxide synthase (eNOS) uncoupling—impairs nitric oxide bioavailability, and activates pro-inflammatory NF-κB pathways, in addition to direct protein damage via N-homocysteinylation (9–11). In stark contrast to the wealth of data on Hcy, the specific mechanisms of MMA-induced vasculopathy are poorly understood, representing a significant knowledge gap. However, based on its known biochemistry, MMA and other accumulated organic acids may further exacerbate vascular injury by directly damaging vascular smooth muscle and the elastic lamina, leading to vessel wall fragility and stiffening (12, 13), and are hypothesized to act synergistically through mitochondrial dysfunction, amplifying oxidative stress and overwhelming cellular defenses (14). This “two-hit” model, in which homocysteine initiates endothelial injury and MMA potentiates it, likely underlies the vascular phenotype. In addition, the confinement of hemorrhages to the cerebellar vermis, despite widespread cerebellar signal abnormalities, suggests region-specific vascular vulnerability or metabolic stress gradients. Notably, the absence of hypertension, coagulopathy, or thrombocytopenia in our patient supports a primary metabolic etiology. Furthermore, beyond hemorrhage, the extensive microangiopathy caused by MMA-HC—including retinopathy—can progress to symptoms such as renal failure, hepatic dysfunction, cardiomyopathy, and pulmonary hypertension (15).

Previous descriptions of CNS hemorrhage in MMA are scarce and differ fundamentally from this case. Prafull Dave et al. reported catastrophic parenchymal hemorrhages in infants during acute metabolic crises, associated with rapid neurological decline and high mortality (8). In contrast, our adolescent patient developed punctate microhemorrhages during clinical recovery, detectable only via high-sensitivity SWI. This temporal dissociation from peak metabolic derangement implies that vascular damage may evolve subacutely or represent a sequela of acute injury. No prior studies have described SWI-detectable microhemorrhages in adolescent or adult MMA-HC, nor have they implicated the cerebellar vermis as a predilection site.

The detection of microhemorrhages in this case underscores the critical advantage of high-field (7.0 T) MRI with SWI sequences in uncovering subtle vascular pathology. Lower-field MRI or standard sequences may lack the susceptibility contrast needed to visualize such lesions. This suggests that microhemorrhages in MMA-HC may be more common than previously recognized but remain underdiagnosed with routine imaging protocols. Targeted SWI imaging during acute/subacute phases—especially in patients with cerebellar symptoms—could enhance detection and provide prognostic insights.

In conclusion, the identification of cerebellar microhemorrhages expands the neurophenotype of late-onset MMA-HC and has practical implications: Acute cerebellar presentation with restricted diffusion may mimic inflammatory (e.g., ADEM) or vascular disorders. Rapid metabolic screening (plasma homocysteine, urinary organic acids, acylcarnitine profile) is critical for early diagnosis. While lesions may resolve radiologically (as in our patient), microhemorrhages imply enduring microangiopathy, potentially increasing long-term risks for cerebrovascular events.

## Data Availability

The original contributions presented in the study are included in the article/Supplementary Material, further inquiries can be directed to the corresponding author/s.
